# Delivery of a nutritional prescription by enteral tube feeding in children with chronic kidney disease stages 2–5 and on dialysis—clinical practice recommendations from the Pediatric Renal Nutrition Taskforce

**DOI:** 10.1007/s00467-020-04623-2

**Published:** 2020-07-29

**Authors:** Lesley Rees, Vanessa Shaw, Leila Qizalbash, Caroline Anderson, An Desloovere, Laurence Greenbaum, Dieter Haffner, Christina Nelms, Michiel Oosterveld, Fabio Paglialonga, Nonnie Polderman, José Renken-Terhaerdt, Jetta Tuokkola, Bradley Warady, Johan Van de Walle, Rukshana Shroff, on behalf of the Pediatric Renal Nutrition Taskforce

**Affiliations:** 1 The Biomedical Research Centre at Great Ormond Street Hospital for Children NHS Foundation Trust and Institute of Child Health, University College Londonfig, WC1N 3JH, London, UK; 2 University of Plymouth, Plymouth, UK; 3 Great Northern Children’s Hospital, Upon Tyne, Newcastle, UK; 4 Southampton Children’s Hospital, University Hospital Southampton NHS Foundation Trust, Southampton, UK; 5 University Hospital Ghent, Ghent, Belgium; 6 Emory University and Children’s Healthcare of Atlanta, Atlanta, USA; 7 Children’s Hospital, Hannover Medical School, Hannover, Germany; 8 PedsFeeds LLC, University of Nebraska, Lincoln, USA; 9 Emma Children’s Hospital, Amsterdam University Medical Center, Amsterdam, The Netherlands; 10 Fondazione IRCCS Ca’Granda Ospedale Maggiore Policlinico, Milan, Italy; 11 British Columbia Children’s Hospital, Vancouver, Canada; 12 Wilhelmina Children’s Hospital, University Medical Center Utrecht, Utrecht, The Netherlands; 13 Children’s Hospital and Clinical Nutrition Unit, University of Helsinki and Helsinki University Hospital, Helsinki, Finland; 14 Children’s Mercy, Kansas City, USA

**Keywords:** Enteral tube feeding, Nasogastric tube, Gastrostomy, Guidelines, Growth, Nutrition

## Abstract

The nutritional prescription (whether in the form of food or liquid formulas) may be taken orally when a child has the capacity for spontaneous intake by mouth, but may need to be administered partially or completely by nasogastric tube or gastrostomy device (“enteral tube feeding”). The relative use of each of these methods varies both within and between countries. The Pediatric Renal Nutrition Taskforce (PRNT), an international team of pediatric renal dietitians and pediatric nephrologists, has developed clinical practice recommendations (CPRs) based on evidence where available, or on the expert opinion of the Taskforce members, using a Delphi process to seek consensus from the wider community of experts in the field. We present CPRs for delivery of the nutritional prescription via enteral tube feeding to children with chronic kidney disease stages 2–5 and on dialysis. We address the types of enteral feeding tubes, when they should be used, placement techniques, recommendations and contraindications for their use, and evidence for their effects on growth parameters. Statements with a low grade of evidence, or based on opinion, must be considered and adapted for the individual patient by the treating physician and dietitian according to their clinical judgement. Research recommendations have been suggested. The CPRs will be regularly audited and updated by the PRNT.

**Electronic supplementary material:**

The online version of this article (10.1007/s00467-020-04623-2) contains supplementary material, which is available to authorized users.

## Introduction

In 2008, the National Kidney Foundation Disease Outcomes Quality Initiative (KDOQI) produced extensive guidelines on all aspects of the nutritional management of children with chronic kidney disease (CKD) stages 2 to 5 and on dialysis (CKD2-5D), including delivery of the nutritional prescription [1]. KDOQI recommends that in the event of inadequate dietary intake and failure to grow at the appropriate rate, the diet should be optimized by nutritional supplementation with energy and/or protein. The prescription should be offered initially by mouth. If there is ongoing failure to thrive, enteral tube feeding is advocated. There is no new evidence to change these guidelines. Indeed, the PRNT CPRs for energy and protein requirements also recommend that supplemental or exclusive enteral tube feeding should be commenced in children who are unable to meet their nutritional requirements orally [2]. However, there is considerable variation worldwide in the adoption of these guidelines. For example, in a study from the International Pediatric Peritoneal Dialysis Network (IPPN) of growth in children under 2 years of age on peritoneal dialysis (PD), tube feeding was predominantly used in North America and Europe, with very little usage elsewhere in the world [3]. Similarly, in a second IPPN study in older children, enteral tube feeding was rarely applied in Central Europe, Turkey, India, South East Asia, and China [4].

KDOQI did not guide readers in the type of enteral feeding device to choose. The European Society for Paediatric Gastroenterology, Hepatology and Nutrition (ESPGHAN) has stated that gastrostomy is the standard of care for all children on long-term enteral tube feeding and has written guidelines on the use of percutaneous endoscopic gastrostomy (PEG) [5]. This advice is echoed in the American Society for Parenteral and Enteral Nutrition (ASPEN) Enteral Nutrition Practice Recommendations [6]. In these PRNT guidelines, we have addressed the benefits and disadvantages of feeding devices in infants and children with CKD2-5D, with particular attention to the special needs of children on dialysis.

## Methods

The composition of the PRNT and the full development process for the CPRs and their purpose, search criteria, grading of evidence, and plans for audit and revision of the CPRs have been previously described [2, 7]. In addition, a pediatric surgeon and interventional radiologist, both highly skilled in the insertion of different enteral feeding devices, provided expert advice on specific aspects of this CPR.

### The PICO questions

The PICO (Patient, Intervention, Comparator and Outcome) format was used to address the questions within the CPR [8]. Our PICO terms were:

*Population*: Children from birth to 18 years of age with CKD2-5D

*Intervention*: Delivery of nutrition by nasogastric (NG) tube or gastrostomy device

*Comparator*: Delivery of nutrition by the oral route, or no comparator

*Outcomes*: Change in weight and height standard deviation score (Wt SDS and Ht SDS) in children with CKD2-5D; and early and long-term complications of gastrostomy feeding, with particular reference to children on PD

### Literature search

Existing papers and guidelines on methods of delivery of the nutritional intake in children with CKD were reviewed and used to make up-to-date recommendations for children with CKD2-5D. Details of papers from the literature search are described in Supplementary Table 1, and Tables 1 and 2. There are no randomized controlled trials (RCTs) of the effects of supplemental feeding in CKD, or of the relative benefits in delivery of the nutritional prescription by the oral route, NG tube, or gastrostomy device. All studies are observational, and most are retrospective. In many, it is not possible to separate out the effects of age, route of delivery, and CKD stage. Most importantly, although the aims of the prescription are provided, the nutritional composition of the formula delivered through the tubes is not described. Because of the lack of high-quality studies, we have included all studies with findings relevant to outcomes, irrespective of patient numbers or duration of follow-up. CPRs are graded as suggested by the American Academy of Pediatrics (Supplementary Table 2) [39].

**Table 1:** Effects of dietary supplementation, enteral feeding and specialist input by a dietician on growth

Study [reference]	No. of patients	Age (year)	GFR (mL/min/1.73 m^2^)	Diet	No. with nasogastric/no. with gastrostomy	Duration (months)	Growth
**Improvement in growth overall**							
Guillot 1980 [9]	3	< 3		Energy 116% EAR, protein 84%	3/0	4–37	2 improved growth, 1 did not
Strife 1986 [10]	3	1.7–3.0	20–25	Starting with 50 kcal/kg and increasing	3/0	11–16	WtVel from < 5 to > 95%, HtVel from 5–40 to 80–95%
Rees 1989 [11]	16	0.2–1.8	7–37	100% RDA energy for CA, 100% RDA protein for Ht age	HtSDS improved in half 10/16 were tube fed: 6 improved their HtSDS. 60% of tube fed improved, 33% improved without tube	60	HtSDS − 2.9 (− 4.1 to − 1.5) At 1 year − 2.6 (− 4.3 to − 0.3) At 2 years − 2.4 (− 4 to 0) At 3 years − 2.0 (− 3.5 to − 0.1) at 5 years − 2.2 to − 0.2)
Brewer 1990 [12]	14	3 days to 3.1	PD	> 90% RDA energy for Ht age, 3–4 g/kg protein	13/0	3–33	Increase in HtSDS and WtSDS in 11
Claris-Appiani 1995 [13]	2 3	< 2 > 2	CRF	101–115% EAR energy, 75–113% protein	5/0	12	ΔHtSDS + 1.56, WtSDS + 1.72
Ledermann 1999 [14]	26 9	0–2 2–5	29 with GFR of 6–26, 6 on PD	100% EAR energy for CA, 100% RNI protein for Ht age with protein supplement for PD	20/9 8 Nissen	24	0–2-year group WtSDS − 3.1 to − 1.7 at 1 year, − 1.4 at 2 years, HtSDS − 2.9 to − 2.2 to − 2.1 2–5-year group WtSDS − 2.3 to − 2 at 1 year, − 1.1 at 2 years, HtSDS − 2.3 to − 2.0 to − 2.0
Kari 2000 [15]	24 13	0.3–2	< 20 PD	100% EAR energy for CA, 100% RNI protein for Ht age with protein supplement for PD	13/17 14 Nissen	≥ 24	HtSDS − 2.34 to − 1.93 HtSDS − 2.17 to − 1.24
Ledermann 2000 [16]	20	< 1	PD	100% EAR energy for CA, 100% RNI protein for Ht age with protein supplement for PD	10/8 7 Nissen	1–59	WtSDS − 1.6 at start, − 0.3 at 1 year, 0.3 at 2 years HtSDS − 1.8 to − 1.1 to − 0.8
Parekh 2002 [17]	17 7	< 1	< 65, polyuric PD	100–160 kcal and 2–2.5 g protein/kg/day, 2–4 mEq sodium, 0.3–0.5 kcal/mL	Both ? ratio	12–24	ΔHtSDS + 1.37 at 1 year, + 1.82 at 2 years
Norman 2004 [18]	25 21	2–16	51–75 25–50	Intensive input from dietician and supplementation if EAR energy and RNI micronutrients < 80%	0/1	24	All on supplements increased Ht and WtSDS No change in HtSDS if GFR > 50 HtSDS + 0.1 SD, correlation between intake and ΔHtSDS if GFR < 50
Hijazi 2009 [19]	52	4.4 range (0.5–18)	PD			1983–1995 *N* = 23 1996–2008 *N* = 29	Only data: − 3 ± 1.5 early era − 1.4 ± 0.9 second era
Mekahli 2010 [20]	101	0.3 (0–1.5)	< 20	100% EAR energy for CA, 100% RNI protein for Ht age with protein supplement for PD	Age at start of enteral feeds 0.8 years (0–4.9) At stop 2.5 (0.1–8.7) years 66% tube fed, 37% gastrostomy 13% Nissen	Up to 20 years	HtSDS (SD) − 0.42 (2.34) at birth (*n* = 40), − 2.07 (1.34) at 0.5 years (*n* = 57), − 1.93 (1.38) at 1 year (*n* = 72), − 1.14 (1.14) at 5 years (*n* = 67), − 1.04 (1.15) at 10 years (*n* = 62), − 1.84 (1.32) at 15 years (*n* = 40), − 1.68 (1.52) at 18 years of age (*n* = 32)
Sienna 2010 [21]	102	1.7 (0.9–15.6)	13.8 (3.9–61.8)		20 tube fed 82 demand fed	2.9 (0.9–11.8)	Mean BMISD (SD) – 1.22 ± 1.68 at start, 0.43 ± 0.86, at removal, 0.68 ± 1.23 at 5 years. Mean HtSDS 2.35 ± 1.86, – 1.51 ± 0.99, – 1.58 ± 1.64 Mean WtSDS − 2.53 ± 1.85, – 0.66 ± 0.97, – 0.16 ± 0.84 Controls over previous 5 years: Mean BMI (SD) 0.30 ± 1.47, 0.23 ± 2.62 Mean HtSDS 1.04 ± 1.38, – 1.17 ± 1.16 Mean WtSDS – 0.33 ± 1.48, – 0.30 ± 1.97 36% of the non-tube fed and 50% of those tube fed were overweight or obese, associated with steroid post-transplant
Rees 2011 [3]	153	< 2	PD		From diagnosis to last observation, 57 patients were fed on demand, 54 by NG and 10 by gastrostomy, 26 switched from NG to gastrostomy and 6 returned from NG to demand feeding		Median Δ BMISDS/year − 0.54 (1.91) during demand feeding vs + 0.97 (3.43) during NG tube feeding, + 1.24 (3.24) during gastrostomy feeding. Median Δ HtSDS/year − 1.35 (2.63) during demand feeding, − 0.72 (1.59) during NG tube feeding, − 0.50 (2.47) during gastrostomy feeding (*p* < 0.05 for gastrostomy vs. demand feeds)
Marlais 2019 [22]	50	2.1–10.9	1 HD, 15 PD 34 GFR 6–88	100% EAR energy for CA, 100% RNI protein for Ht age with protein supplement for PD	5 NG, 40 gastrostomy	24	Overall HtSDS improved from − 2.39 to − 2.27 at 1 year and − 2.18 after 2 years (*p* = 0.02). BMISDS − 0.72 to 0.23 after 1 year and 0.09 after 2 years (*p* < 0.0001). HtSDS improved more in children aged 2–6 years (− 2.13 to − 1.68, *p* = 0.03) and in children not on dialysis (− 2.33 to − 1.99, *p* = 0.002)
**No change in growth rate**							
Ramage 1999 [23]	8 7	< 2.5 > 2.5	PD	Aiming for 100% RNI energy or greater if no response	0/12	12	Decline in HtSDS arrested No change
Coleman 1999 [24]	13	0.2–8.5	PD	5.9 and 3.1 dietician contacts/month	1/7	36	HtSDS from − 1.2 to − 1.14 and WtSDS from − 1.32 to − 0.73
Shroff 2003 [25]	18	0.5–2	HD	100% EAR energy for CA, 100% RNI protein for Ht age with protein supplement for PD	9/8	0.3–26	No change in HtSDS or WtSDS
Abitbol 1993 [26]	12	0.25–2	< 70	100% RDA energy, > 140% protein	9/3	Up to 24	Ht and WtVel lowest (− 2SD) by 6 months and Ht and WtSDS by 12 months of age, then stable at − 2SD
**Deterioration in growth**							
Reed 1998 [27]	7	0.6 ± 0.7	Mean 17 (< 30)	Energy based on median Wt for CA	7/0	18.6 ± 4.5	HtSDS − 0.9 to − 1.1, WtSDS − 0.4 to − 0.2
Ellis 2001 [28]	137	< 5	PD/HD	Not specified	Equal at < 2 years, 29%/57% at 2–5 years	Until age 6 years	WtSDS − 0.77 at start, − 2.04 at 1 year, HtSDS − 1.75 and − 2.89 without supplements, WtSDS − 1.33 and − 1.70 and HtSDS − 1.76 and − 2.88 with supplements, respectively
**Benefits of specialist input by a dietician**							
Norman 1998 [29]	2	0.25	PD	11.8 dietetic contacts/month predialysis, 8.4 during the first year on PD, 4.3 during the second year	Both gastrostomy	2	HtSDS increased from − 1.66 to − 0.17 and 0.67 to 0.78, WtSDS from − 1.26 to − 0.43 and 0.31 to 1.75
Coleman 1999 [24]	781 dietetic contacts during 182 patient-months	7.7 (range 0.2 to 8.5)	PD	Children < 5 years 5.9 (1.9) contacts/patient/month vs 3.1 (1.6) in children > 5 years of age	82% of contacts were with children receiving nutritional support via a button		Mean Ht and WtSDS were − 1.2 and − 1.32 at the start, and at the end were − 1.14 and − 0. 73, and BMISDS from − 0.91 to 0. 17

*BMI*, body mass index; *CA*, chronological age; *CRF*, chronic renal failure; *EAR*, estimated average requirement; *GFR*, glomerular filtration rate; *HD*, hemodialysis; *Ht*, height; *NG*, nasogastric; *PD*, peritoneal dialysis; *RDA*, recommended daily allowance; *RNI*, reference nutrient intake; *SD(S)*, standard deviation (score); *Vel*, velocity; *Wt*, weight

**Table 2:** Effects of gastrostomy tube placement on infection risk

Study [reference]	Patients—number and age (year)	Intervention and comparator groups	Outcome—peritonitis rates	Outcome—PD technique survival/patient survival
Balfe 1990 [30]	*N* = 20 Age range 0.3 to 12.8	PD patients who were enterally fed – 13 G-tubes (10 were on PD prior to the G-tube insertion) No comparator	G-tube exit site infections in 4, one of these patients developed peritonitis No details on technique of G-tube insertion	Not stated
Coleman 1998 [31]	*N* = 22 Age range 0.2 to 10.3	*n* = 14 children with G-tube and PD catheter inserted simultaneously vs. *n* = 2 with G-button and PD catheter simultaneously vs. *n* = 2 with G-tube after initiating PD (+ 4 on HD)	2 patients had peritonitis attributed to G-tube (includes 1 fungal peritonitis in a malnourished patient) Open gastrostomy recommended	Not stated
Ramage 1999 [32]	*N* = 23 Mean age 3.8 ± 3.2	2 groups: 1. G-tube inserted prior to the commencement of PD (*n* = 9; includes *n* = 2 who also underwent surgical G-tube replacement while receiving PD) 2. G-tube inserted while receiving PD (*n* = 14) Type and technique of G-tube insertion—not stated Controls—children on chronic PD without a G-tube (*n* = 127)	Peritonitis occurred every 18.4 patient-months in controls and 7.8 patient-months in those with a G-tube (*p* < 0.001) Peritonitis occurred every 6.0 patient-months before and 8.1 patient-months after G-tube insertion in those undergoing G-tube insertion on PD	PD catheter replacement secondary to infection occurred every 109.4 patient-months in controls and 39.9 patient-months in those with a G-tube Peritonitis did not occur before G-tube insertion and occurred every 32.5 patient-months following G-tube insertion
Ledermann 2002 [33]	*N* = 29 Median age 3.9 (0.5–13.3)	3 groups: 1. G-tube (PEG/open G-tube ± Nissen) *before* starting PD (*n* = 15) 2. Open G-tube ± Nissen *after* PD catheter insertion/start of PD (*n* = 9) 3. PEG *after* PD catheter insertion/start of PD (*n* = 5)	Group 2 (130 months G and PD), 0.2 vs. 1.4 episodes of peritonitis/patient-year before and after G-tube insertion Group 3 0.5 episodes of peritonitis/patient-year before and 4 out of 5 children developed peritonitis soon after PEG placement	In group 3 (PEG after PD) - 2 transferred to hemodialysis, 1 remained on PD after treatment of *Candida* peritonitis and 1 died
Rahim 2004 [34]	*N* = 68 Mean 9 (range 0.1 to 19)	Presence of G-tube (*n* = 68) vs. no G-tube (*n* = 59) G-tube before PD cath *n* = 41 G-tube at the same time as a PD catheter *n* = 13 G-tube after PD catheter *n* = 14 G-tube insertion technique—not stated	Presence of a G-tube was associated with a higher peritonitis rate (*p* < 0.001) compared to chronic PD without a G-tube No difference in the peritonitis rate between patients with a G-tube inserted before or after a PD catheter (*p* = 0.46) No difference in the peritonitis rate between patients with a G-tube inserted before or at the same time as a PD catheter (*p* = 0.06)	Not stated Note—some patients were switched from PD to HD after G-tube placement for approximately 4–6 weeks while the G-tube tract healed (no clear indications or difference in outcomes with this practice mentioned)
von Schnakenburg 2006 [35]	*N* = 27 Median 1.3 (range 0.25 to 10.9) years	G-tube insertion after Tenchkoff catheter insertion/patient already on PD 25 PEG and 2 open gastrostomies No comparator group	Early peritonitis < 7 days after PEG in 10/27 (37%); was effectively treated with intraperitoneal antibiotics in 4/10 Fungal peritonitis in 7/27 (26%) patients *Note* • significant variations in practice across sites. Variations in antibiotic and antifungal prophylaxis used • most patients were malnourished	In those with fungal peritonitis: • 4 cessations of PD and change to hemodialysis • 2 deaths In 18/27 (67%) patients, PD was successfully reinitiated shortly after PEG insertion
Lindley 2012 [42]	*N* = 33 Mean age 4.9 in OPEN group and 3.7 years in LAP group	2 groups: 1. laparoscopic-assisted PEG and PD catheter insertion (*n* = 10) – LAP group 2. open gastrostomy and PD catheter (*n* = 23) – OPEN group	Peritonitis and infection rates* per catheter-year were 0.89 and 0.7 in LAP and 0.59 and 0.5 in OPEN group (not significant) The risk of peritonitis and infection was not related to method of placement (not significant)	PD catheter survival - median 12 months in the LAP group and 17 months in the OPEN group (not significant)
Phan 2013 [36]	*N* = 7 Median 10	*n* = 207 on chronic PD *n* = 7 (3%) with concomitant gastrostomies Timing and technique of G-tube insertion - not stated	Not stated	Hazard ratio for re-operations for infections was 5.01 (95% CI 1.5–16.6) higher in children with gastrostomies compared to those without gastrostomies, *p* = 0.008 (on fully adjusted multivariable model)
Prestidge 2015 [37]	*N* = 17 7.2 (range 10 weeks to 17.2 years)	14/17 (82%) open surgical technique, 3 laparoscopic A G-tube was inserted in 15 patients after PD had been established 2 patients had simultaneous G-tube and PD catheter insertion	- 2 cases of early peritonitis with organisms derived from the gastrointestinal tract - no case of fungal peritonitis No statistically significant difference between incident rates of bacterial peritonitis before G-tube placement (0.6 episodes per patient-year; 95% CI 0.26–1.18) and post-G-tube placement (1.21 episodes per patient-year; 95% CI 0.69–1.97)	- No PD technique failure - No deaths
Zaritsky 2018 [38]	*N* = 156 infants with chronic PD catheter placed prior to their first birthday 53% under 30 days at PD catheter insertion	3 groups: - G-tube placement before or at the same time as PD catheter (*n* = 47) - G-tube placement after PD catheter insertion/start of PD (*n* = 49) - No G-tube (*n* = 61)	G-tube insertion after catheter placement was associated with a nearly 3-fold [OR (95% CI) 2.81 (1.31, 6.01), *p* < 0.01] increased risk of peritonitis	Not stated

*Peritonitis was defined as the presence of a white blood cell count > 100/mm3 with at least 50% being polymorphonuclear leukocytes, and infection was defined as the presence of positive peritoneal cultures with peritonitis

*CI*, confidence interval; *G*, gastrostomy; *HD*, hemodialysis; *PD*, peritoneal dialysis; *PEG*, percutaneous endoscopic gastrostomy; *OR*, odds ratio

Given the very low grade of evidence for most recommendations, we conducted a Delphi survey (e-questionnaire) involving an international group of expert dietitians and pediatric nephrologists as described previously [2, 7].

## Clinical practice recommendations

1. When should enteral tube feeding be commenced?
  1.1 We suggest that supplemental or exclusive enteral tube feeding should be commenced in children who are unable to meet their nutritional requirements orally, in order to improve their nutritional status. (grade B, strong recommendation)
  1.2 We suggest that there should be prompt intervention once deterioration in weight centile is noted. (grade B, strong recommendation)

### Evidence and rationale

The causes of poor nutritional intake in CKD and the adverse effects of this on growth are well-described [9, 40]. However, studies of benefits of enteral feeding suffer from deficiencies such as mixing of ages, CKD stages, and inappropriate or lack of comparator groups. In addition, details of the type of formula administered via the enteral feeding device, and concurrent use of recombinant human growth hormone (rhGH) are rarely provided. These factors cause difficulties when assessing and comparing outcomes.

Studies are summarised in Table 1 [3, 10–29], and divided according to whether there has been an improvement, no change, or a deterioration in growth. The majority of studies are in the very young. This is to be expected as it is in the infantile phase that dependency of growth on nutrition is at its maximum and if inadequate, can result in the loss of as many as two Ht SDS [21, 40]. A benefit to growth was seen in seven [3, 12, 16–18, 21, 23] of the ten [3, 12, 16–18, 21, 23, 26–28] studies in those under 2 years of age. In the largest study of 153 infants on PD, growth was better preserved in association with gastrostomy than demand or NG feeding [3]. The high potential for declining height velocity in infancy means that it is logical to address nutritional intake and failure to grow at this time without delay. Assessment of the benefits of enteral feeding after 2 years of age is more difficult to assess as many studies contain a mixture of ages, or include children who begin the study in the infantile phase of growth. However, prompt intervention is recommended at all ages: during the childhood phase of growth, benefit was seen in nine [10, 11, 13–15, 19, 20, 22, 23] of the 13 studies [10, 11, 13–15, 19, 20, 22–26, 29].

Two studies have estimated the number of contacts with a dietician required to achieve a growth benefit (Table 1). They emphasized the need for more frequent contact when treating the youngest patients, which was estimated to be as high as 13 contacts per month, but was still high at 5 contacts per month for those over 5 years of age [24, 41].

2. What are the optimal feeding devices for short-term and long-term enteral feeding?
  2.1 An NG tube is the preferred option for short-term enteral feeding, and may be considered as a bridging option to a long-term enteral feeding tube. (ungraded)
  2.2 A gastrostomy device is preferable to an NG tube for long-term enteral feeding. (ungraded)
  2.3 The enteral feeding device for long-term management should be determined in partnership between the parents/caregivers and healthcare team. (ungraded)

### Evidence and rationale

NG tubes are useful for early nutritional support in infants, until, if long-term support is required, an acceptable body weight for gastrostomy placement is reached, as determined by individual units. Insertion of an NG tube is a simple procedure which is easy to teach, and there is no risk of peritonitis in children on PD as a result of NG tube usage. However, there are disadvantages to NG tubes. They are easily displaced and the trauma of frequent replacement has adverse effects on both the child and the caregiver. There are reports of inhibition of the development of oral motor skills and subsequent speech and swallowing problems. They can cause sinusitis, otitis media, and nasoseptal erosion. They give a visible message that the child is a “sick child,” which can affect normal psychosocial development. Most importantly, they are associated with vomiting and therefore are associated with an increased risk for aspiration [42]. Displacement and formula inhalation, which can be fatal, is a risk if an NG tube is used to provide an unsupervised, continuous overnight feed in a home environment.

ESPGHAN and ASPEN guidelines state that a gastrostomy is the standard of care for long-term enteral feeding, avoiding all the complications of an NG tube [5, 6]. However, gastrostomies have associated complications as well, including tube malfunction, and skin breakdown from leakage. Rare complications, such as injury to adjacent organs, inadvertent creation of a gastrocolic fistula during insertion, intra-abdominal leakage, and peritonitis, are also reported [5, 42]**.**

Adequate time should be allocated for discussion with the parents and caregivers so that they have a good understanding as to why a gastrostomy is required, and the process of tube feeding. Informed consent, including discussion of potential complications, should be obtained by the operating surgeon. As for all invasive procedures, the child must be adequately prepared, preferably by a trained play therapist/child life specialist. Peri-operative care should also include carer education on how to use the gastrostomy, and discussions on how oral intake will be encouraged and when they might expect the gastrostomy could be removed [43].

3. What preparations should be made prior to insertion of a gastrostomy device? What are the techniques used for the insertion of gastrostomy devices?

3.1 Investigations such as an upper gastrointestinal contrast study, esophageal impedance, or pH studies prior to gastrostomy device placement may be considered on an individual patient basis. (grade D, weak recommendation)
3.2 Gastrostomy devices can be placed as a percutaneous endoscopic gastrostomy (PEG), percutaneous radiologically inserted gastrostomy (RIG), open surgical, or percutaneous laparoscopic-assisted gastrostomy (PLAG). (ungraded)

### Evidence and rationale

Although there are is no evidence to support the routine use of contrast studies before placement of a gastrostomy, many surgeons request this to evaluate the anatomy of the stomach and duodenum [5]. Many children with CKD have gastro-esophageal reflux disease (GERD) and vomiting despite optimizing anti-reflux medications. This may improve with small, frequent feeds through the gastrostomy and, for severe cases, can also be dealt with by converting the gastrostomy tube to a gastro-jejunal tube [40, 42]. The requirement to undertake studies to evaluate for GER needs to be decided on an individual basis [40].

Gastrostomy devices may be inserted percutaneously and endoscopically (PEG), by radiological guidance (RIG) or surgically. The reader is referred to reviews of the placement techniques for these devices [5, 30, 42]. All gastrostomy devices directly enter the stomach through the abdominal wall; differences are in the methods of insertion and the types of fixation. Fixation within the stomach may be with an internal disc, flange, or a water-filled balloon. Devices with an internal disc or flange last longer than those with a balloon, but changing these tubes is often uncomfortable and may require sedation or anesthesia. Gastrostomies held in place by a balloon can be inserted primarily or after maturation of a gastro-cutaneous tract following insertion of a gastrostomy tube such as a Malecot. These gastrostomies, unlike those with a disc or flange, can be changed easily and without discomfort, usually every 6 months or so. All gastrostomies, regardless of fixation in the stomach, can be opened and attached directly to the tube delivering the feed.

4. What patient characteristics determine which gastrostomy insertion technique should be used?
  4.1 A PLAG or open gastrostomy is the preferred procedure in patients already receiving PD. (grade C, strong recommendation)
  4.2 We suggest that in a child who is likely to need PD, and in whom enteral tube feeding is required, gastrostomy tube insertion by PEG or RIG should, whenever possible, be performed before placement of a PD catheter. (grade C, strong recommendation)
  4.3 A PLAG or open gastrostomy are the preferred procedures for patients who have had previous abdominal surgery, and those with severe kyphoscoliosis, gastric ulcers, or varices. (grade C, weak recommendation)

### Evidence and rationale

An important difference in gastrostomy insertion techniques, which is of particular relevance to children on PD, is that with the PEG and RIG techniques, the gastric wall is not stitched to the abdominal wall, increasing the risk of leakage of stomach contents into the peritoneum. In contrast, during laparoscopic or open surgery, the stomach is sewn directly to the abdominal wall, which decreases this risk. PEG can be combined with laparoscopic visualisation as another alternative (PLAG). It has been suggested that PLAG combines the benefits of the creation of a tight fit around the exit site from the PEG technique with suturing of the stomach to the abdominal wall to prevent leakage of gastric contents into the peritoneum, but there are no comparative studies to assess this [30].

There are 11 studies of gastrostomy and PD-related infection. They differ in the technique and timing of gastrostomy insertion, use of a comparator group, use of antibiotic and/or antifungal prophylaxis at the time of surgery, and outcome measures. These studies are summarized in Table 2 [31–38, 44–46]. Four studies [39,40,42,46], although retrospective and with small numbers, suggest an up to 3-fold higher risk [45] for bacterial and fungal peritonitis and PD failure when a PEG tube is inserted in a child already established on PD. Of surgical techniques, there are two studies that have shown no difference in peritonitis rates between PLAG placement and an open surgical procedure [36, 46]. In one study, risks were significantly higher irrespective of whether the gastrostomy was placed before commencement of PD or while the child was receiving PD compared with age-matched children on PD without gastrostomy devices [32]. However, other small single center studies do not show differences in peritonitis rates based on the type of gastrostomy device or the technique of insertion [31, 32, 36].

Given these data, and the potentially devastating consequences of peritonitis, the PRNT conclude that, whenever possible, a gastrostomy device should be inserted prior to the placement of a PD catheter, and, in a child established on PD, an open surgical placement or PLAG be performed. This is the view expressed by the International Society for Peritoneal Dialysis (ISPD), which also recommends that in the child receiving PD, insertion of gastrostomy should be by an open procedure [47]. It is important that if a PD catheter is placed in a child who might need a gastrostomy in the future, the PD exit site is not positioned over the stomach. Attention to peri-operative antibiotics is critical for patients with a PD catheter in place who require placement of a gastrostomy tube (and also for placement of a PD catheter in a patient who has a gastrostomy in place). These patients should have pre-operative dosing of antibiotics which cover skin flora, as well as antifungal coverage [47].

Units vary in their practice regarding the lowest body weight at which a gastrostomy placement will be considered. There are reports of successful gastrostomy tube insertion in infants weighing as little as 2.1 kg, with no increase in the complication rate in comparison to older children [48]. Infants may require an open technique and may be too small for most button gastrostomies, requiring placement of the more classical tube gastrostomy.

Relative contraindications for endoscopic placement are kyphoscoliosis or abdominal surgery, which may distort the position of intra-abdominal organs; and ulcers or varices, when a PEG tube insertion increases the risk of bleeding [5]. It has been suggested that insertion of a PEG might also cause new peristomal varices and *de novo* portosystemic shunts, increase the risk of splenic injury in those with splenomegaly, as well as complicate surgery for a future liver transplant [5, 49, 50]. However, neither the ESPGHAN nor the French Society for Gastrointestinal Endoscopy comment on open gastrostomy insertion in these patients [5, 51]. There are only two studies of 7 patients describing PEG-related complications in children with portal hypertension [52, 53]. In an international web-based survey of pediatric nephrologists, hepatologists, and surgeons, 92% of respondents agreed that the benefits of gastrostomy feeding outweighed associated theoretical risks and complications in children with autosomal recessive polycystic kidney disease [54].

Transpyloric tubes are recommended by ESPGHAN only in circumstances of severe GER disease, gastroparesis or gastric outflow obstruction [5]. Positioning of the tube within the jejunum is crucial and insertion requires radiological guidance. Nasojejunal tubes are easily displaced; hence, gastro-jejunal tubes are an alternative. Both gastro-jejunal and nasojejunal tubes require that all formula delivery is by continuous infusion, as boluses entering the jejunum are poorly tolerated.

5. Is a gastrostomy device associated with an increased risk of peritonitis in the long-term?
  5.1 We suggest strict attention to the care of the exit sites of the gastrostomy and PD catheter to help prevent exit site infections and cross infection. (grade B, moderate recommendation)

### Evidence and rationale

Studies have reported a significantly higher rate of PD exit site infections, peritonitis and PD catheter replacement in children on PD who are gastrostomy fed [31–35, 37]. This does mean that clinicians need to weigh up the relative risks and benefits of NG and gastrostomy feeding. The higher peritonitis rate is reported even several months after gastrostomy or PD catheter insertion, implying that careful long-term care of both exit sites is important. PD and gastrostomy exit sites may be in close proximity, so good exit site care is important to prevent cross infection [47]. PD exit site infection can lead to organisms tracking down the catheter subcutaneous tunnel, leading to peritonitis. The increased peritonitis rate did not extend to fungal infections [55]. Peritonitis rates were not related to the type of gastrostomy device or the technique of insertion [31, 32, 36] (Table 2). It is logical to try to distance the PD and gastrostomy exit sites as far as possible.

6. Can a gastrostomy device be inserted at the same time as a PD catheter?
  6.1 We suggest that a gastrostomy device can be inserted simultaneously with a PD catheter if the gastrostomy is placed by PLAG or open surgery. (grade B, strong recommendation)

### Evidence and rationale

There is one retrospective study of PD catheter placement and simultaneous PLAG or surgically placed gastrostomy; peritonitis rates and PD catheter survival did not differ for the two insertion techniques [36]. Similarly, no difference in peritonitis rate was seen with simultaneous PD catheter and gastrostomy insertion compared with gastrostomy insertion before PD catheter [35] (Table 2). ISPD also recommends that gastrostomy placement should preferentially be performed either before or at the time of PD catheter placement [47].

7. What precautions should be taken to prevent peri- and post-operative complications of gastrostomy tube placement in the child on PD?
  7.1 Antibiotic prophylaxis, based on local antibiotic sensitivities, is recommended for all children undergoing gastrostomy placement. (grade C, strong)
  7.2 We recommend that children who are already established on PD or who receive a gastrostomy at the same time as a PD catheter receive broad spectrum antibiotic and antifungal prophylaxis in the peri-operative period of gastrostomy placement. (grade C, strong)
  7.3 We suggest that PD should be withheld for 24 h or longer after gastrostomy placement if it is clinically safe to do so. (ungraded)

### Evidence and rationale

A Cochrane meta-analysis of adults (not on PD) undergoing PEG placement showed that prophylactic antibiotics decreased gastrostomy exit site infection [56]. However, in a single-center retrospective study of children, again not on PD, antibiotic prophylaxis did not decrease the risk of infectious complications [57]. Amongst children on PD, the lowest rate of infectious complications after PEG placement was observed in patients who received both antifungal and antibiotic prophylaxis [42]. Malnourished patients and those on acid blocking medications have a higher risk of fungal infection when a gastrostomy is placed [58].

Given the high dextrose content of dialysate and the potential for bacterial contamination of the peritoneum at the time of gastrostomy placement, we strongly recommend antibiotic and antifungal prophylaxis to lower the risk of peritonitis in children already on PD at the time of gastrostomy insertion. On the basis of a Cochrane review [56], ESPGHAN [5, 59] recommend pre-operative antibiotic prophylaxis to reduce stomal infection rates, but do not comment on the type, timing or duration of antibiotic prophylaxis either for children on PD, or when PD and gastrostomy tubes are inserted simultaneously. ISPD [47] guidelines recommend using a single dose of cefazolin (or vancomycin if MRSA risk is high). Given the risk of peritoneal contamination with both cutaneous as well as enteric pathogens, broad spectrum antibiotic covering enteric pathogens and an antifungal (fluconazole is commonly used) is suggested. The choice of antibiotic prophylaxis will depend on local antimicrobial sensitivity patterns.

The antibiotic and antifungal agents should be given just prior to initiation of the surgical procedure: ISPD guidelines strongly recommend that peri-operative antibiotic prophylaxis be used within 60 min before the incision for PD catheter placement [47]. The duration of treatment is not defined in any study, but 48 h is considered reasonable in children on PD, given that there is a possibility of leakage of gastric contents during and after gastrostomy insertion, until an adequate seal has formed between the stomach and abdominal wall.

There are no studies on the optimum time that should be allowed to elapse before PD is recommenced after gastrostomy placement. ISPD guidelines recommend 24 h [47]. It would be logical to stop the infusion of dialysate for at least 24 h, although the patient remains at risk until the seal around the stomach and abdominal wall has healed, which may take 48–72 h. It is also logical to reduce the usual fill volumes and build them up slowly over several days to prevent both peri-wound leakage and pain. Children who receive CAPD may benefit from CCPD while hospitalized, until normal fill volumes can be resumed. The time over which reduced dialysis can be maintained will depend on the presence of residual kidney function, fluid and dietary intake and laboratory results. The nutritional prescription may need to be modified in response to volume overload and rising levels of urea, potassium and phosphate resulting from reduced dialysis. There is no evidence to suggest that there needs to be a switch from PD to HD during gastrostomy insertion.

8. When and how should enteral tube feeding be started?
  8.1 We suggest cautious introduction of a water bolus (after discussion with the insertion operator), followed by the gradual introduction of formula over the next 6 h. (ungraded)

### Evidence and rationale

There is no evidence for when tube feeding can be safely started after insertion of a gastrostomy device, or the type or rate of formula to be given. The consensus of the PRNT is to commence introduction of water via the gastrostomy, usually 2 h after placement. ESPGHAN suggests that feeding can be commenced four to 6 h post-PEG placement but these recommendations do not include children on PD [5]. Experience from a single large center (Great Ormond Street Hospital for Children, London) suggests that a small volume of water (e.g., 30 ml, scaled to the child’s size) given 2 h post-insertion of gastrostomy device is safe. If this has been tolerated, then at 3 h, 50% of the usual feed volume is given as water, changing to 50% of the usual feed volume as formula 6 h post-insertion and 100% at 7 h, so that by 8 h, the usual feeding regimen can be restarted, taking into account any necessary changes to the formula composition resulting from the period of nil by mouth and reduced dialysis. A similar approach is appropriate in a child starting enteral tube feeding for the first time.

9. How should the formula be delivered using the enteral feeding tube?

9.1 Tube feeding may be exclusive or supplementary to oral feeding. The method of feeding, rate and volume should be discussed with the family. (ungraded)
9.2 To encourage the continuation of oral intake during the day, all the tube feed, or a portion of it, may be given by continuous infusion overnight. (grade D, weak recommendation)
9.3 Continuous infusion feeding may be beneficial if vomiting is a problem. (ungraded)
9.4 NG tubes must only be used with close supervision in the home environment, as there is a significant, although rare, risk of aspiration, which can be fatal. (grade X)

### Evidence and rationale

The approach for enteral tube feeding should take into consideration the individual child and their home circumstances. Supplementary formula can be given by enteral tube after meals or to complete a scheduled feeding of oral formula, or given as a daytime bolus, either by gravity or administered more slowly via an enteral feeding pump. Delivery of all, or a majority, of the formula overnight via an enteral feeding pump may promote hunger and an interest in oral food and fluid intake during the day.

Parents should be encouraged and supported to maintain their child’s oral motor skills, despite the fact that some children require all of their nutrition to be provided through enteral feeding. Long-term tube feeding can disrupt normal feeding development and milestones. Even if a child will not feed from a bottle, pureed and more textured foods should be offered when they are developmentally ready. Parents can offer positive oral experiences such as gently touching their child’s mouth or cheeks and kissing them, giving a pacifier or bottle to suck, encouraging licking or tasting foods without any pressure to chew or swallow them, or letting them mouth toys. The older child can be encouraged to engage in messy play with food, self-feed by hand or with a spoon, prepare foods and join the family at the table at mealtimes. Educating parents on the importance of avoiding force-feeding is crucial [30]. Prompt identification and management of feeding problems is vital to minimize food refusal, achieve some oral intake and smooth the transition to full oral intake. Families may require support from the multidisciplinary team, such as the dietitian, clinical psychologist, play therapist, speech therapist or occupational therapist [43, 60–62]. Strategies for oral stimulation and prevention of dependency on enteral tube feeding are described elsewhere [43, 60, 61]. Given the frequent presence of decreased appetite and gastrointestinal motility in children with CKD2-5D (associated with elevated circulating levels of polypeptide hormones and cytokines) [9, 40], successful transition from tube feeding to oral diet may not happen until after transplant. Appetite stimulants are not recommended in the general pediatric population as an intervention for children dependent on tube feeds [62], and the PRNT does not recommend these for children with CKD either.

The slow delivery of formula by continuous infusion via an enteral feeding pump can be beneficial in those with more severe GER and may reduce vomiting [42]. However, there is a significant risk of aspiration, which can be fatal, if an NG tube is used unsupervised for continuous overnight feeding at home.

10. How should vomiting be managed if it is affecting growth despite medical therapy and continuous gastrostomy feeding?

10.1 We suggest evaluation for gastro-esophageal reflux if vomiting continues in association with gastrostomy feeding and affects growth. Upper gastrointestinal contrast and pH studies are needed to exclude malrotation and to define the severity of gastro-esophageal reflux, respectively. Placement of a gastro-jejunostomy or Nissen fundoplication may be needed. (grade D, weak recommendation)

### Evidence and rationale

There is no evidence that gastrostomy placement can induce GER and, in fact, there is some evidence that gastrostomy feeding may reduce or prevent vomiting [4]. An NG tube may stent open the gastro-esophageal junction, increasing any tendency to vomiting and GERD [3]. Indications for further intervention are no different in children with CKD than other children. ESPGHAN recommends an anti-reflux procedure for erosive esophagitis or GER with an unsafe swallow [5]. As for any surgical procedure on the abdomen in a child on PD, this requires careful planning and either temporary suspension of PD or conversion to hemodialysis.

11. When can a child transition from tube to oral feeding?
  11.1 If the child develops an interest in taking food by mouth, we suggest decreasing the nutrition provided by tube feeding in proportion to oral intake, provided an adequate rate of growth is maintained. The goal is for the child to feed orally to meet nutritional goals. (grade D, weak recommendation)

### Evidence and rationale

Whereas long-term tube feeding does not preclude the development of normal eating and drinking and successful transition from tube to oral feeding, the time taken to transition to complete oral feeding has varied in the literature from 2 to 10 months. The evidence is in the post-transplant population when the transition from tube feeding typically occurs [17,35,61,62,64]. The feeding regimen may be altered in the following way to encourage eating after transplant: reduction of the feed volume initially by 25% to promote an appetite for food; moving from continuous to bolus feeds to coincide with a more normal eating pattern; and setting a daily target for eating, with bolus supplements of the nutritional deficit via tube after meals where intake is below target.

Plans for the transition from enteral to oral feeding should be individualized according to the child’s feeding skills and behaviors. Children who commenced enteral feeding in the first 2 years of life may not have developed normal oral motor patterns in relation to feeding, whereas children who had eaten prior to being tube fed may need to be “re-taught” how to eat [60]. “Hunger-inducing” programs for weaning children off enteral tube feeds have been described [62], but these are not recommended for children with CKD. Even if the child achieves an adequate intake of food, the enteral tube may necessary to administer sufficient volumes of fluid and medications, particularly after transplant. When a decision is made that a gastrostomy is no longer needed, the removal procedure depends on the type and longevity of the device. All tubes that are held in place by an internal phalange need surgical removal. A button can be removed and the tract may close spontaneously, but if the tract is well established, surgery may be needed to close it [42].

## Results of the Delphi survey

The Delphi survey was sent to 49 pediatric nephrologists and 40 dietitians. Of these 35 pediatric nephrologists and 31 dietitians from 21 countries returned a completed survey, a 58% overall response rate. The names of all respondents are listed under “Acknowledgments” below.

Of the 22 clinical practice recommendation statements, 17 of 22 statements achieved above 70% agreement from all respondents. Analyzing the level of agreement for each statement, overall an 81% consensus was achieved with a “strongly agree or agree” response and a 16% “neutral” response, reflecting the wide variations in practice in the absence of robust evidence. The highest “disagree or strongly disagree” rate was in response to statement 7.2 on antibiotic and antifungal prophylaxis in the peri-operative period of gastrostomy placement in the child on PD. There was disagreement on the need for antifungal prophylaxis and whether broad spectrum antibiotics were needed (as opposed to antibiotics that cover skin flora only), although most authors disagreed on the basis of personal practice rather than published evidence. On careful review of the literature and discussion within the Taskforce team it was agreed that even though it may be a rare event, the prevention of peritonitis, particularly fungal peritonitis, and the potential loss of peritoneal membrane function requiring a change of dialysis modality, both broad spectrum antibiotics and antifungal medications was appropriate. Based on suggestions from Delphi respondents, minor re-wording of two statements and further clarification to the text was done.

## Summary of recommendations

A summary of recommendations is provided in Table 3.

**Table 3:** Summary of recommendations

Question		Recommendation	Grading of evidence
1. When should enteral tube feeding be commenced?	1.1	We suggest supplemental or exclusive enteral tube feeding should be commenced in children who are unable to meet their nutritional requirements orally, in order to improve their nutritional status.	Grade B, strong recommendation
	1.2	We suggest that there should be prompt intervention once deterioration in weight centile is noted.	Grade B, strong recommendation
2. What are the optimal feeding devices for short-term and long-term enteral feeding?	2.1	An NG tube is the preferred option for short-term enteral feeding, and may be considered a bridging option to a long-term enteral feeding tube.	Ungraded
	2.2	A gastrostomy device is preferable to an NG tube for long-term enteral feeding.	Ungraded
	2.3	The enteral feeding device for long-term management should be determined in partnership between the parents/caregivers and healthcare team.	Ungraded
3. What preparations should be made prior to insertion of a gastrostomy device? What are the techniques used for the insertion of gastrostomy devices?	3.1	Investigations such as an upper gastrointestinal contrast study, esophageal impedance or pH studies prior to gastrostomy device placement may be considered on an individual patient basis	Grade D, weak recommendation
	3.2	Gastrostomy devices can be placed by percutaneous endoscopic gastrostomy (PEG), percutaneous radiologically inserted gastrostomy (RIG), open surgical, or percutaneous laparoscopic-assisted gastrostomy (PLAG).	Ungraded
4 What patient characteristics determine which gastrostomy insertion technique should be used?	4.1	A PLAG or open gastrostomy is the preferred procedure in patients already receiving PD.	Grade C, strong recommendation
	4.2	We suggest that in a child who is likely to need PD, and in whom enteral tube feeding is required, gastrostomy tube insertion by PEG or RIG should, wherever possible, be performed before placement of a PD catheter.	Grade C, strong recommendation
	4.3	A PLAG or open gastrostomy are the preferred procedures for patients who have had previous abdominal surgery, or who have severe kyphoscoliosis, and gastric ulcers or varices.	Grade C, weak recommendation
5. Is a gastrostomy device associated with an increased risk of peritonitis in the long-term?	5.1	We suggest strict attention to care of exit sites of the gastrostomy and PD catheter to help prevent exit site infections and cross infection.	Grade B, moderate recommendation
6. Can a gastrostomy device be inserted at the same time as a PD catheter?	6.1	We suggest that a gastrostomy device can be inserted simultaneously with a PD catheter if the gastrostomy is placed by PLAG or open surgery.	Grade B, strong recommendation
7. What precautions should be taken to prevent peri- and post-operative complications in the child on PD?	7.1	Antibiotic prophylaxis, based on local antibiotic sensitivities, is recommended for all children undergoing gastrostomy placement.	Grade C, strong
	7.2	We recommend that children who are already established on PD or who receive a gastrostomy at the same time as a PD catheter receive broad spectrum antibiotic and antifungal prophylaxis in the peri-operative period of gastrostomy placement.	Grade C, strong
	7.3	We suggest that PD should be withheld for 24 h or longer after gastrostomy placement if it is clinically safe to do so.	Ungraded
8. When and how can enteral tube feeding be started?	8.1	We suggest cautious introduction of a water bolus (after discussion with the insertion operator), followed by gradual introduction of feeds over the next 6 h.	Ungraded
9. How can the feed be delivered using the enteral feeding tube?	9.1	Tube feeding may be exclusive or supplementary to oral feeding. The method of feeding, rate and volume should be discussed with the family.	Ungraded
	9.2	To encourage the continuation of oral intake during the day, all the tube feed, or a proportion of it, may be given overnight.	Grade D, weak recommendation
	9.3	Continuous infusion feeding may be beneficial if vomiting is a problem.	Ungraded
	9.4	NG tubes must only be used with close supervision in the home environment, as there is a significant risk of aspiration, which can be fatal.	Grade X
10. How should vomiting be managed if it is affecting growth despite medical therapy and continuous gastrostomy feeding?	10.1	We suggest evaluation for gastro-esophageal reflux if vomiting continues in association with gastrostomy feeding and affects growth. Upper GI contrast and pH studies are needed to exclude malrotation and to define the severity of gastro-esophageal reflux, respectively. Nissen fundoplication may be needed.	Grade D, weak recommendation
11. When can a child transition from tube to oral feeding?	11.1	If the child develops an interest in taking food by mouth, we suggest decreasing the nutrition provided by tube feeding in proportion to oral intake, provided an adequate rate of growth is maintained. The goal is for the child to feed orally to meet nutritional goals.	Grade D, weak recommendation

## Research recommendations

1. To investigate the relationship between feeding via gastrostomy device and vomiting pattern by comparing no tube, NG feeding, and gastrostomy.
2. To determine the barriers to gastrostomy device placement in healthcare providers and caregivers of children with CKD who have inadequate oral nutritional intake.
3. To investigate changes in quality of life outcomes of caregivers and patients after gastrostomy device placement.
4. To investigate interventions that optimize the transition from tube feeding to oral feeding.
5. To determine the optimal technique for gastrostomy placement in established PD patients.
6. To describe the effect of changes in rate and volume of continuous and bolus feeds on the frequency and volume of emesis.
7. To determine the amount and frequency of consultation with the dietitian supervising the nutritional prescription to effect a positive outcome on growth.

## Electronic supplementary material

ESM 1 (DOC 389 kb)
