# Supplementary material for: Delivery of a nutritional prescription by enteral tube feeding in children with chronic kidney disease stages 2–5 and on dialysis—clinical practice recommendations from the Pediatric Renal Nutrition Taskforce
Source: Pediatr Nephrol. 2020 Jul 29;36(1):187–204. doi: 10.1007/s00467-020-04623-2 (PMC7701061; doi:10.1007/s00467-020-04623-2)

**Supplementary Material**

**Delivery of a nutritional prescription by enteral tube feeding in children with chronic kidney disease stages 2-5 and on dialysis – clinical practice recommendations from the Pediatric Renal Nutrition Taskforce**

**Supplementary Table 1 Search terms strategy used in the literature review**

1980 – October 2019; English language

Medline, PubMed, Embase, Cochrane library, Cinahl, manual searching

Meta-analyses, randomised controlled trials, prospective studies in patients with CKD and on dialysis (prospective studies in children only included)

Retrospective pediatric studies in children with CKD and on dialysis

| 1 | **kidney disease** | **renal failure** | **renal insufficiency** | **chronic kidney disease** | **kidney failure** | **kidney injury** | **kidney dysfunction** | **CKD** |
| --- | --- | --- | --- | --- | --- | --- | --- | --- |
|  | **CRF** | **CKF** | **ESRD** | **ESRF** | **dialysis** | **renal replacement therapy** | **pre dialysis** | **peritoneal dialysis** |
|  | **hemodialysis** | **haemodialysis** | **CAPD** | **APD** |  |  |  |  |
| **2** | **Gastrostomy tube** | **Percutaneous endoscopic gastrostomy** | **PEG tube** | **Radiological inserted gastrostomy (RIG)** | **RIG tube** | **Open gastrostomy** | **Surgical gastrostomy** |  |
| **3** | **Nasogastric tube** | **NG tube** |  |  |  |  |  |  |
|  |  |  |  |  |  |  |  |  |

**Supplementary Table 2** **American Academy of Pediatrics grading matrix**


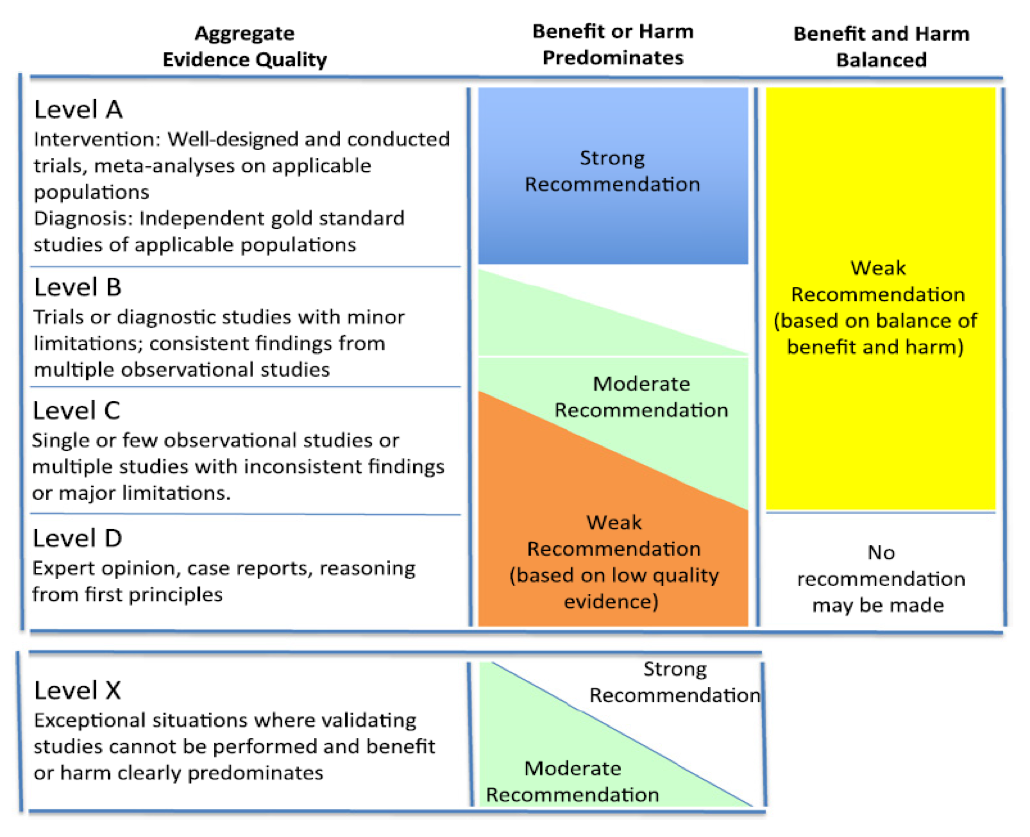

Supplement: Supplementary file 1 — (DOC 389 kb) [file 467_2020_4623_MOESM1_ESM.doc]
